# Efficacy of Some Botanical Extracts against *Trogoderma granarium* in Wheat Grains with Toxicity Evaluation

**DOI:** 10.1100/2012/639854

**Published:** 2012-04-19

**Authors:** Aly S. Derbalah

**Affiliations:** Pesticides Department, Faculty of Agriculture, Kafr El-Sheikh University, Kafr El-Sheikh 33516, Egypt

## Abstract

In an attempt to find alternative control methods for stored products insects, extracts of seven plant species (*Cassia senna, Caesalpinia gilliesii, Thespesia populnea var. acutiloba, Chrysanthemum frutescens, Euonymus japonicus, Bauhinia purpurea*, and *Cassia fistula*) were evaluated under laboratory conditions for their ability to protect wheat (*Triticum* spp.) grains against *Trogoderma granarium* insect. Moreover, gas chromatography-mass spectrometry (GC-MS) analysis was carried to identify the chemical components of the most effective plant extract against *T. granarium*. Furthermore, the safety of the most effective plant extract was evaluated with respect to biochemical and histological changes in treated rats relative to control. The results revealed that, the tested botanical extracts showed high efficiency against *T. granarium* with respect to mortality and progeny of the adults. *C. senna* was the most effective botanical extract against *T. granarium*. The GC-MS analysis of the most effective plant extract showed the presence of different bioactive compounds that is known by its insecticidal activity. The most effective plant extract showed no toxicity on treated rats relative to control with respect to biochemical and histological changes. The results suggest the ability of using these plant extracts for wheat grains protection as a safe alternative to insecticides.

## 1. Introduction

Stored products of agricultural and animal origin are attacked by more than 600 species of beetle pests, 70 species of moths, and about 355 species of mites causing quantitative and qualitative losses [1], and insect contamination in food commodities is an important quality control problem of concern for food industries. In industrialized countries like Canada and Australia there is zero tolerance for insects in food grains [2]. *T. granarium* is among the most serious and of widest occurrence in stores in tropical and subtropical regions of Asia and Africa [3] and is common in geographical areas characterized by high temperature and low humidity [4]. 

Control of stored-product insect populations is primarily dependent upon continued applications of insecticides [2]. In spite of its efficacy, their repeated use for several decades has disrupted biological control system by natural enemies and led to outbreaks of insect pests, widespread development of resistance, undesirable effects on nontarget organisms, and environmental and human health concerns [2, 5].

These problems have highlighted the need for the development of new types of selective insect-control alternatives. Plants may provide potential alternative to currently used insect-control agents because they constitute a rich source of bioactive chemicals [6]. Since these are often active against a limited number of species including specific target insect, they are often biodegradable to nontoxic products, potentially suitable for use in integrated pest management, and they could lead to the development of new classes of safer insect-control agents. Much effort has, therefore, been focused on plant-derived materials for potentially useful products as commercial insect-control agents. Little work has been done to manage stored-product insects by using aromatic medicinal plants despite their excellent pharmacological actions [7, 8].

Most of the alternatives insecticides substances were tested against insects attacking stored products in order to establish new control practices with lower mammalian toxicity and lower persistence in the environment relative to insecticides. Therefore, studies should conduct not only on the evaluation of botanical extracts against the target pests but also on their safety on human health that are in demand. Although the assessment of enzymes activity in the blood is generally a more sensitive measure of compound toxicity than histopathological changes and can be assessed within a shorter time, the tissue alterations are considered a confirmatory and supporting diagnostic role in the case of certain abnormalities in blood sampling [9].

Therefore, this study attempted to evaluate insecticidal activity of some newly used plant extracts (*C. senna*, *C. gilliesii*, *T. populnea var. acutiloba*, *C. frutescens*, *E. japonicus*, *B. purpurea*, and *C. fistula*) against* T. granarium *in wheat grains with respect to progeny and mortality of the insect adults, to identify the chemical components of the most effective plant extract against* T. granarium*, and finally to evaluate the toxicity of the most effective plant extract on rats with respect to biochemical and histological changes relative to control.

## 2. Materials and Methods

### 2.1. The Insect

*T. granarium *(Everts) was obtained from the Department of Stored Product Pests Control, Research Institute of Plant Protection, Sakha, Kafr El-Shiekh. This strain was reared free of insecticidal contamination for several years at  30 ± 2°C and 70% ± 5 relative to humidity. The cultures were maintained under the same conditions in the Pesticide Department, Faculty of Agriculture, Kafr El-Shiekh University, Egypt. The culture was raised by infesting 30 pairs of newly emerged* T. granarium *adults into 500 g of wheat grains in large box. After that, 35 d newly emerged (F1) adults were collected and used to infest the wheat samples.

### 2.2. The Stored Product

Wheat grains were used to culture *T. granarium* and to evaluate the efficacy of tested plant extracts as well as malathion against the same insect as well. Wheat grains were stored in airtight tins until being required for experiments. The experiments were carried out in a room kept at a constant temperature of 25°C and 70% r.h.

### 2.3. Plants and Preparation of Crude Extracts

The leaves of seven medicinal plant species (*C. senna*, *C. gilliesii*, *T. populnea var. acutiloba*, *C. frutescens*, *E. japonicus*, *B. purpurea*, and *C. fistula*) were collected from a local nursery at Kafr El-Sheikh, Monofia, Gharbia, and Alexandria Governorates, Egypt. *C. senna* (Alexandrian Senna), belonging to the family Fabaceae, is native to tropical Africa and cultivated in Egypt and Sudan. *C. gilliesii* (bird of paradise), belonging to the family Fabaceae, is native to tropical America, mainly Argentina and Uruguay. *T. populnea var. acutiloba* (*Portia Tree*), belonging to the family Malvaceae, is native to South Africa. *C. frutescens* (marguerite daisy), belonging to the family Asteraceae, is native to the Canary Islands. *E. japonicus* (Japanese Spindle), belonging to the family Celastraceae, is native to Japan, Korea, and China. *B. purpurea* (Purple camel's foot), belonging to the family Fabaceae, is native to South China. *C. fistula* (Cassias), belonging to the family Fabaceae, is native to southern Asia. The different leave samples were oven dried for 24 h at 70°C and, then, finely powdered using a blender. Each sample (25 g) was extracted twice with 300 mL of methanol at room temperature for 2 days. The extracts were filtered through Whatman filter paper (no. 15), Whatman Inc. (North Americ) USA. The combined filtrate was concentrated to dryness by rotary evaporation at 40°C.

### 2.4. Effect of Tested Plant Extracts and Malathion on Progeny of *T. granarium*

The tested plant extracts at concentration levels of 100, 300, and 500 ppm were used to evaluate its efficacy against* T. granarium*. Malathion was used as recommended compound against *T. granarium* at concentration levels of 5, 10, and 20 mg/L. Each concentration was applied in three replicates, and each replicate contained 20 g of wheat grains. The treatment of wheat grains was carried out by dipping wheat grains in water solution of malathion and botanical extracts at tested concentration levels twice consecutively for 5 seconds and subsequently spread on top of plastic sheets to dry for 90 min. The control treatment was carried using water only and replicated three times. Then, 10 adults of *T. granarium* were transferred to treated wheat grains which were put in a 85 × 75 mm plastic jar and kept at  30 ± 2°C and 70% ± 5 r.h, according to the method described by Kestenholz et al. [10]. The emerged adults from the hatched eggs were recorded after 6 weeks of treatment. These adults were used to calculate the reduction percentages in* T. granarium* progeny from the use of the tested plant extracts as well as malathion compared to the control as shown in the following equation as described by El-Lakwah et al. [11]:

(1) $$\begin{array}{cc} \text{\%}\;\text{Reduction} & =\frac{\text{MNEC}-\text{MNET}}{\text{MNEC}}\times 100,\\ \text{MNEC} & =\text{Mean}\;\text{no}.\;\text{of}\;\text{those}\;\text{which}\;\text{emerged}\;\;\\ & \;\text{in}\;\text{the}\;\text{control},\\ \text{MNET} & =\text{No}.\;\text{of}\;\text{those}\;\text{which}\;\text{emerged}\;\text{in}\;\text{the}\;\\ & \;\text{treatment}. \end{array}$$

### 2.5. Efficiency of the Tested Plant Extracts and Malathion on Adults, Pupae, and Larvae of *T. granarium* Beetle by Mean Mortality

Wheat grains were treated with the tested plant extracts and malathion for protection against larvae, pupae, and adults of *T. granarium* at concentration levels mentioned before. Each concentration was applied in three replicates and in each replicate contained 20 gm of wheat grains. The treatment of wheat grains was carried out by dipping wheat grains in aqueous solution of malathion and botanical extracts at the tested concentration levels twice consecutively for 5 seconds and subsequently spread on top of plastic sheets to dry for 90 min. The control treatment was carried using water only and replicated three times. Then, 10 adults, pupae, and larvae of *T. granarium* were transferred to treated wheat grains which were put in a 85 × 75 mm glass jar and kept at  30 ± 2°C and 70% ± 5 r.h, according to the method described by Kestenholz et al. [10]. The glass jars were covered with cotton cloths held on with rubber bands. The number of dead adults, pupae, and larvae in each jar was counted after one and two weeks and the percentage of insect mortality was recorded.

### 2.6. Chemical Composition of the Most Effective Plant Extract

GC/MS analysis was carried to identify the components of the most effective plant extract* (C. senna*) according to the method described by Durate-Almeida et al. [12]. The samples were injected three times for confirmation. The analysis was conducted on HP 6890 GC system coupled with a 5973 network mass selective detector with a capillary column of HP-5MS (60 m × 0.25 mm, film thickness 0.25 m). The oven temperature program was turned on at 50°C, held for 2 min, and then raised up to 200°C at a rate of 5°C·min^−1^. Helium was used as the carrier gas at a flow rate 1.0 mL·min^−1^, with a split ratio equal to 1/50. The detector and injector temperatures were 250 and 200°C, respectively. Some of the detected compounds in the tested plant extracts were identified by comparison of their retention indices (RIs) and mass spectra fragmentation with the available analytical standards (1,8 Cineole, Linalool, and Butanoic acid). They were also identified by comparison of their RIs and mass spectra fragmentation with those stored in the Wiley and NIST libraries associated with GC-MS. Several other compounds could be identified only through the second method. The samples were analyzed by the Central Laboratory for Pesticides, Agriculture Research Centre, Cairo, Egypt.

### 2.7. Toxicity Assessments

#### 2.7.1. Animal Treatment

The used adult Wistar male rats *(Rattus norvegicus*) with 8 weeks old and 80–100 gm in weight were obtained from Faculty of Medicine, Tanta University. Wister rats were housed in wire cages under standard conditions with free access to drinking water and food. The rats were kept in temperature-controlled room with 14 hours light and 10 hrs dark cycles. The rats were given a standard diet as described by Romestaing et al. [13]. Before treatment, rats were left two weeks for adaptation. The animals were randomly divided into two groups each comprising of three animals one group for the treatment with the most effective plant extract (*C. senna*) till 21 days and the second group for control. The most effective plant extract was administered to rats orally at concentration level of 500 mg/kg body weight. Control group rats were orally administrated with equal amount of almond oil. After 21 days the rats were sacrificed under anesthesia. Then, the blood samples were taken by cardiac puncture in vials containing heparin. Moreover, specimens from kidney and liver were taken from each treatment and kept in neutral buffered formalin 10% for histopathological test.

#### 2.7.2. Enzymes Assays

Blood samples were centrifuged at 4500 rpm for 15 min at 4°C and the blood serum was used to determine the Glutamate Pyruvate Transaminase (GPT), creatinine, and alkaline phosphatase (ALP) according to the methods described by Barham and Trinder [14], Reitman and Frankel [15], and Wilkinson et al. [16], respectively.

#### 2.7.3. Histopathological Test

The histopathology test was carried out at Histopathology Laboratory, Department of Histopathology, Faculty of Veterinary Medicine, Kafr El-Sheikh University, according to the method described by Bancroft and Stevens [17].

### 2.8. Statistical Analysis

Data from the experiments were statistically analyzed using one-way repeated measurement analysis of variance. For mortality experiments, the statistical analysis was carried out after mortality percentages were corrected. Newman-Keuls's multiple range test using a computer program SAS (Version 6.12, SAS Institute Inc., Cary, NC, USA) was used to separate means.

## 3. Results

### 3.1. Effect of Tested Plant Extracts and Malathion on Progeny of *T. granarium*

The numbers of emerged adults of *T. granarium* were significantly decreased in all treatments (the tested plant extracts and malathion) relative to the control, as shown in Table 1. Moreover, the tested plant extracts delayed the progeny of the tested insect three weeks relative to control treatment. Increasing the concentration level of all tested treatments reduced the emergence of *T. granarium* even more (concentration dependent).* C. senna* extract followed by malathion and *B. purpurea* was the most effective treatment while *C. furtescens* extract was the least effective one.

### 3.2. Efficiency of Tested Plant Extracts and Malathion on *T. granarium* Adults Determined by Mortality Values

The efficacy of the tested plant extracts and malathion against *T. granarium* adults by means of mortality was presented in Table 2. The results showed that* C. senna* was the most effective treatment against *T. granarium* adults followed by *B. purpurea*, *C. gilliesii*, *E. japonicus*, *T. populnea var. acutiloba*, *C. fistula*, malathion, and *C. frutescens*, respectively. The morality percentages of *T*. *granarium *were significantly increased in the second week relative to the first week at all tested treatments. Increasing the concentration level of all tested treatments increased the mortality of *T*. *granarium *adults even more (concentration dependent).

### 3.3. Efficiency of Tested Plant Extracts and Malathion on *T. granarium* Pupae Determined by Mortality Values

The efficacy of the tested plant extracts and malathion against *T. granarium* pupae by means of mortality was presented in Table 3. The results showed that* B. purpurea *was the most effective treatment against *T. granarium* pupae followed by *C. senna*, *C. gilliesii*, *E. japonicus*, *T. populnea var. acutiloba*, *C. fistula*, malathion, and *C. frutescens*, respectively. The morality percentages of *T*. *granarium *pupae were significantly increased in the second week relative to the first week at all tested treatments. Increasing the concentration level of all tested treatments increased the mortality of *T. granarium *pupae even more (concentration dependent).

### 3.4. Efficiency of Tested Plant Extracts and Malathion on *T. granarium* Larvae Determined by Mortality Values

The efficacy of the tested plant extracts and malathion against *T. granarium* larvae by means of mortality was presented in Table 4. The results showed that* C. gilliesii *was the most effective treatment against *T. granarium* larvae followed by *E. japonicus*, *C. senna, B. purpurea*, *T. populnea var. acutiloba*, malathion, *C. furtescens*, and *C. fistula*, respectively. Among the tested plant extracts, *C. gilliesii* extract was the most effective one and *C. fistula* extract recorded the lowest efficacy against the larvae of *T*. *granarium*. The mortality percentages of *T*. *granarium *larvae were significantly increased in the second week relative to the first week at all tested treatments. Increasing the concentration level of all tested treatments increased the mortality of *T*. *granarium *larvae even more (concentration dependent).

### 3.5. Composition of the Most Effective Botanical Extract

The identified chemical components of the most effective botanical extract (*C. senna*) against *T. granarium* were presented in Table 5. Eighteen compounds were identified from *C. senna* extract, separately with different percentages as shown in Table 5.The identified compounds were belonging to different fatty acids and their derivatives (eldyhydes, esters, and alcohols).

### 3.6. Toxicity Evaluation

#### 3.6.1. Effect of the Most Effective Plant Extract on Liver Enzymes

The Alkaline phosphatase and GPT activities are known as cytosolic marker enzymes reflecting hepatocellular necrosis as they are released into the blood after cell membrane damage. In the present study, therefore, both enzyme activities were used as indicators of hepatic damage. The obtained data in Table 6 showed that there were no significant differences in the activity of ALT and GPT after 21 days of rat's administration with the most effective plant extract at dose level of 500 mg/kg body weight relative to control treatment.

#### 3.6.2. Effect of the Most Effective Plant Extract on Kidney Function

Regarding the kidney function, there were no significant differences in creatinine level in rats administration with the most effective plant extract at dose level of 500 mg/kg relative to control (Table 6). The normal creatinine in rats treated with the most effective plant extract relative to control treatment was assumed to be the normal kidney function. Moreover, the histology of kidney tissue treated with the most effective plant extract relative to control supports this explanation.

#### 3.6.3. The Histopathological Changes in the Kidney

**Figure 1:**
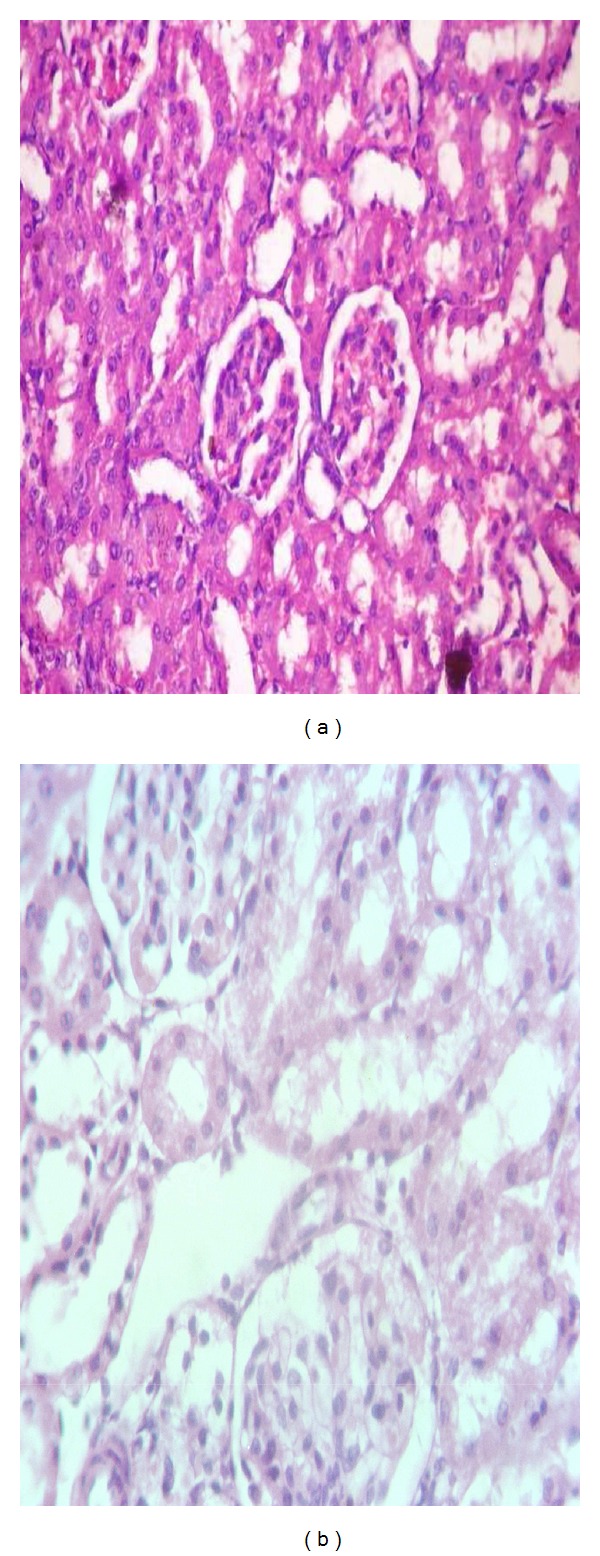
Sections in kidney of rats treated with *C. senna* extract (b) at dose level of 500 mg/kg after 21 days of treatment relative to control (a).

The normal structure of kidney tissue in control treatment was shown in Figure 1(a). However, for the rats treated with *C. senna* extract at dose level of 500 mg/kg, the tissue was some what like control with a small vaculation and degeneration in renal tubules (Figure 1(b)).

#### 3.6.4. The Histopathological Changes in the Liver

**Figure 2:**
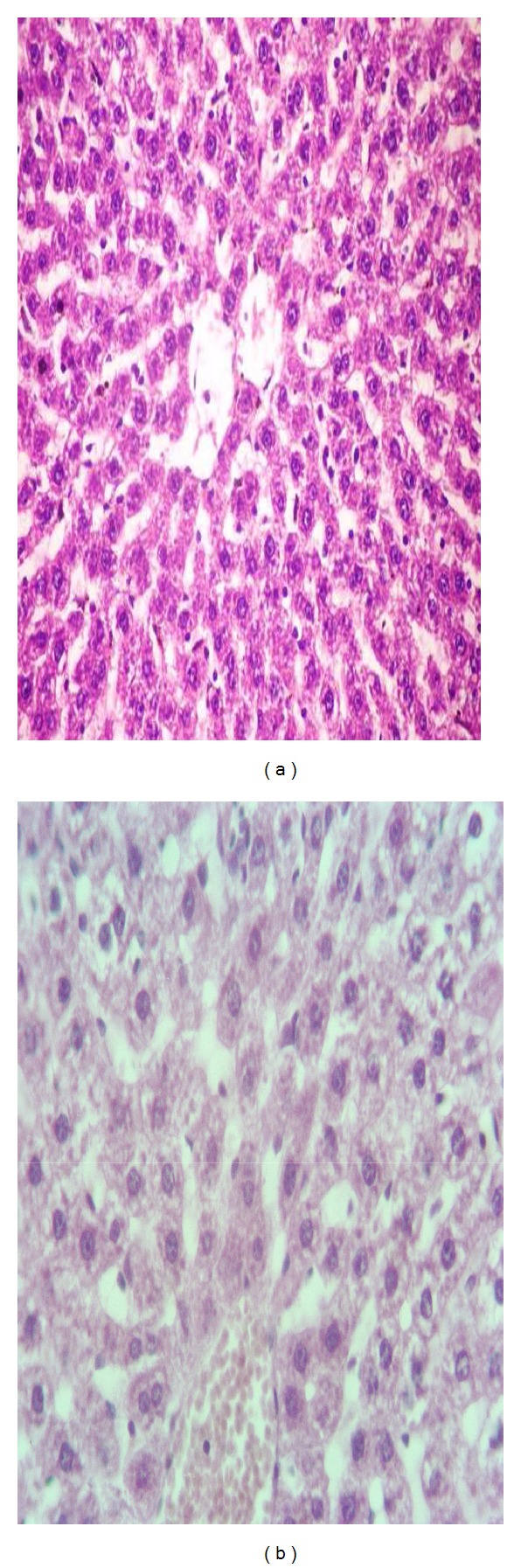
Sections in liver of rats treated with *C. senna* extract (b) at dose level of 500 mg/kg after 21 days of treatment relative to control (a).

The normal structure of liver tissue in control treatment was shown in Figure 2(a). However, the liver of rats treated with *C. senna* at dose level of 500 mg/kg showed that blood vessels engorged and hepatocyte contain vacuolated cytoplasm (Figure 2(b)).

## 4. Discussion

The results of the present study implied that the tested plant extracts were effective against *T. granarium* in stored wheat with respect to progeny of adults and mortality of all its stages (larvae, pupae, and adult). The efficacy of the plant extracts against* T. granarium* insect in stored wheat with respect to progeny and adult mortality has been reported by many researchers [10, 18, 19]. However, the efficacy of the tested plant extracts, especially the most effective ones, has not been reported against* T. granarium* and considered first report.

Among the identified compounds from *C. senna *extract, some compounds such as 1,8 cineole, linalool, butanoic acid, *α*-terpineol acetate, and croweacin were detected with high percentages relative to other detected compounds. The insecticidal activity of *C. senna *extract against *T. granarium* may be due to the presence of the previous fatty acids and its derivatives [20–24]. Moreover, the efficacy of the most effective plant extract at higher concentrations might actually have efficacy comparable to the chemical pesticides. In fact, the actual dosage of any one compound identified in this extract could be relatively low, safe, and economically feasible.

Although the insecticidal activity of the most effective plant extract is attributed mainly to its major compounds mentioned before, the synergistic or antagonistic effect of some compounds in the mixture has to be considered [25]. Each of the plant extract components has its own contribution on biological activity of the extract against the tested insect.

The mode of action of the bioactive natural monoter-penoids (hydrocarbons, alcohols, and ketones) isolated from plant extracts oils may be due to inhibition of acetylcholinesterase [26–28]. Since Lee et al. [28] reported that 1, 8-Cineole was the most potent inhibitor of AChE among the monoterpenes tested. This inhibition may be a mode of action for essential oils and monoterpenes against stored grain insects as well. Also, the mode of action of the tested botanical extracts may be largely attributable to its fumigant action [20, 29].

The botanical extracts as pest control agents present two main characters: the first is their safety to the people and the environment, and the second is the less resistance development against it by the tested insect. Regarding the safety, the toxicity evaluation of the most effective plant extract revealed that there were some slight variations that occurred sporadically in treated rats relative to control with respect to enzyme markers and histopathology of treated organs. Moreover, the observed changes in the tissues were mostly uncorrelated with the dosages which reflect the safety of the tested plant extract on human health. With referring to resistance development, it is believed that it is difficult for the insect to develop resistance to such a mixture of bioactive components with, apparently, different mechanisms of insecticidal activity [30].

This study is considered the first step toward more investigation and concern about using these effective botanical extracts as alternative for controlling of stored product pests. This will help to reduce the environmental pollution and the adverse effect on human health resulted from using insecticides since these botanical extracts revealed nonsignificant toxicity relative to the high dosage that were given orally and will not reach human by this dose as a residue under any conditions.

## 5. Conclusions

The insecticidal activity of the tested plant extracts against *T. granarium* indicated the potential of some plant species (*C. senna*, *B. purpurea*, and* C. gilliesii*) as a natural source of insecticidal material. Insecticidal activity was confirmed in all the tested plant species, although the results showed variation in their effectiveness against *T. granarium* insect. The ability of using botanical products as alternative of chemical control of* T. granarium* is possible if the problem of cost-effective commercial production can be solved. Moreover, some of these botanical extracts could find a place in IPM strategies, especially where the emphasis is on environmental, food safety and on replacing the more dangerous toxic insecticides. Work in this regard should continue to obtain information regarding its practical effectiveness under natural conditions to protect the stored products without any side effects.

## Figures and Tables

**Table 1:** Effect of the tested plant extracts and malathion on progeny of *T. granarium*.

Treatments	Concentration level (mg/L)	% Reduction
*Cassia senna*	100	95^bc^
	300	100^a^
	500	100^a^
	100	82^g^
*Caesalpinia gilliesii*	300	92.5^cd^
	500	97.5^ab^
	100	70^i^
*Thespesia populnea var. acutiloba*	300	90^ed^
	500	92.5^cd^
*Chrysanthemum frutescens*	100	62^j^
	300	70^i^
	500	85^f^
	100	70^i^
*Euonymus japonicus*	300	90^ed^
	500	95^bc^
*Bauhinia purpurea*	100	97.5^ab^
	300	97.5^ab^
	500	100^a^
*Cassia fistula*	100	80^hg^
	300	92.5^cd^
	500	95^bc^
Malathion	5	80^hg^
	10	97^ab^
	20	100^a^
Control	0.0	0.00^k^

^a,b,c,d,e,f,g,h,i,j,k^Separation of means according to the Student Newman Keuls multiple range test (*P* < 0.05).

**Table 2:** Effect of the tested plant extracts and malathion on adult's mortality of *T. granarium*.

Treatments	Concentration level (mg/L)	% Mortality after one week	% Mortality after two week
*Cassia senna*	100	86.7^abc^	100^a^
	300	90^abc^	100^a^
	500	93^abc^	100^a^
	100	43^de^	100^a^
*Caesalpinia gilliesii*	300	60^abc^	100^a^
	500	86.7^abc^	100^a^
	100	40^c^	90^a^
*Thespesia populnea var. acutiloba*	300	63^d^	93^a^
	500	83^bc^	100^a^
*Chrysanthemum frutescens*	100	50^de^	70^b^
	300	60^de^	97^a^
	500	83^bc^	100^a^
	100	60^de^	90^a^
*Euonymus japonicus*	300	80^c^	97^a^
	500	96.7^ab^	100^a^
*Bauhinia purpurea*	100	83^bc^	100^a^
	300	90^abc^	100^a^
	500	93^abc^	100^a^
*Cassia fistula*	100	57^e^	93^a^
	300	63^d^	100^a^
	500	80^c^	100^a^
Malathion	5	60^de^	90^a^
	10	80^a^	93^a^
	2 0	87^a^	100^a^
Control	0.00	0.00^f^	0.00^c^

^a,b,c,d,e,f,g,h,i,j,k^Separation of means according to the Student Newman Keuls multiple range test (*P* < 0.05).

**Table 3:** Effect of the tested plant extracts and malathion on mortality of *T. granarium* pupae.

Treatments	Concentration level (mg/L)	% Mortality after one week	% Mortality after two week
*Cassia senna*	100	66.7^def^	100^a^
	300	86.7^abc^	100^a^
	500	93^bc^	100^a^
	100	43^hi^	97^ab^
*Caesalpinia gilliesii*	300	47^ghi^	100^a^
	500	53^fgh^	100^a^
	100	33^ig^	100^a^
*Thespesia populnea var. acutiloba*	300	60^efg^	100^a^
	500	80^bcd^	100^a^
*Chrysanthemum frutescens*	100	33^jg^	80^de^
	300	47^ghi^	87^bc^
	500	53^fgh^	100^a^
	100	73^cde^	80^e^
*Euonymus japonicas*	300	80^bcd^	93^cd^
	500	83^bcd^	100^a^
*Bauhinia purpurea*	100	80^bcd^	100^a^
	300	93^ab^	100^a^
	500	100^a^	100^a^
*Cassia fistula*	100	33^g^	93^bc^
	300	40^hi^	95^bc^
	500	47^gh^	100^a^
Malathion	5	47^ghi^	67^f^
	10	80^bcd^	93^abc^
	20	93^ab^	100^a^
Control	0.00	0.00^k^	0.00^g^

^a,b,c,d,e,f,g,h,i,j,k^Separation of means according to the Student Newman Keuls multiple range test (*P* < 0.05).

**Table 4:** Effect of tested plant extracts and malathion on mortality of *T. granarium* larvae.

Treatments	Concentration level (mg/L)	% Mortality after one week	% Mortality after two week
*Cassia senna*	100	80^bc^	93^a^
	300	93^ab^	100^a^
	500	97^ab^	100^a^
	100	80^bc^	100^a^
*Caesalpinia gilliesii*	300	93^abc^	100^a^
	500	94^ab^	100^a^
	100	46.6^ef^	87^bc^
*Thespesia populnea var. acutiloba*	300	80^bc^	100^a^
	500	93^ab^	100^a^
*Chrysanthemum frutescens*	100	47^ef^	80^c^
	300	53^e^	87^bc^
	500	60^de^	93^ab^
	100	60^de^	100^a^
*Euonymus japonicus*	300	73^cd^	100^a^
	500	93^ab^	100^a^
*Bauhinia purpurea*	100	33^fg^	93^ab^
	300	53^e^	100^a^
	500	60^de^	100^a^
*Cassia fistula*	100	37^g^	87^bc^
	300	47^ef^	93^ab^
	500	60^de^	100^a^
Malathion	5	50^ef^	87^c^
	10	80^bc^	93^ab^
	20	93^a^	100^a^
Control	0.00	0.00^h^	0.00^d^

^a,b,c,d,e,f,g,h,i,j,k^Separation of means according to the Student Newman Keuls multiple range test (*P* < 0.05).

**Table 5:** The main constituents of *C. senna* plant extract identified by GC-MS analysis.

No.	Name	Retention time (min)	% Area
1	Beta phellandrene	4.33	1.5
2	Mone inositol	4.37	2.75
3	1,8 Cineole	4.98	26.55
4	Linalool	5.70	10.28
5	3 cyclohexen-1-ol 4-methyl-1-(1-methylethyl)	6.57	2.69
6	3cyclohexene-1-methanol-alpha 4-trimethyl-p-menth 1-en-8-ol	6.71	2.48
7	Butanoic acid	7.2	20.02
8	*α*-Terpineol acetate	8.16	21.06
9	9-Octadecanoic acid (z)2, 6 octadien-1-ol 3, 7 dimethyl acetate	8.37	2.2
10	Caryophyllene	8.85	0.75
11	Cycloheptasiloxane tetradecamethyl	9.25	1.7
12	Croweacin	9.72	20.3
13	1, 6 dodecatrien-3-ol 3, 7, 11 titramethyl	9.95	1.12
14	Cyclononosiloxane octadecanmethyl	11.97	2.23
15	Hexadecanoic acid methyl ester	13.07	0.94
16	Tetratetracontane	15.04	1.19
17	Tetra cosamethyl cyclododecasiloxane	19.19	2.6
18	Iron monocarbonyl 1,3 butadiene 1,4, dicarbonic acid diethyl ether	20.13	1.83

**Table 6:** Effect of the most effective plant extract (*C. senna*) on serum GPT, ALT, and creatinine of treated rats at dose level of 500 mg/kg body weight.

Treatments	SGPT U/L	ALP U/L	Creatinine mg/dL
Control	65 ± 1.39	101 ± 5.57	0.205 ± 0.06
*C. senna*	67 ± 3.57	111 ± 5.1	0.197 ± 0.08

## References

[1] Rajendran S, Sriranjini V (2008). Plant products as fumigants for stored-product insect control. *Journal of Stored Products Research*.

[2] White NDG, Leesch JG,  Subramanyam B, Hagstrum DW (1995). Chemical control. *Integrated Management of Insects in Stored Products*.

[3] Viljoen JH (1990). The occurrence of Trogoderma (Coleoptera: Dermestidae) and related species in southern Africa with special reference to *T. granarium* and its potential to become established. *Journal of Stored Products Research*.

[4] Ghanem I, Shamma M (2007). Effect of non-ionizing radiation (UVC) on the development of *Trogoderma granarium* Everts. *Journal of Stored Products Research*.

[5] Subramanyam B, Hagstrum DW, Subramanyam B, Hagstrum DW (1995). Resistance measurement and management. *Integrated Management of Insects in Stored Products*.

[6] Wink M, van Beek TA, Breteler H (1993). Production and application of phytochemicals from an agricultural perspective. *Phytochemistry and Agriculture*.

[7] Tang W, Eisenbrand G (1992). *Chinese Drugs of Plant Origin*.

[8] Namba T (1993). *The Encyclopedia of Wakan-Yaku (Traditional Sino-Japanese Medicines) with Color Pictures, Vol I*.

[9] Cronelius CE, Charles W, Arhode E (1959). Serum and tissue transaminase activities in domestic animals. *The Cornell Veterinarian*.

[10] Kestenholz C, Stevenson PC, Belmain SR (2007). Comparative study of field and laboratory evaluations of the ethnobotanical *Cassia sophera* L. (Leguminosae) for bioactivity against the storage pests *Callosobruchus maculatus* (F.) (Coleoptera: Bruchidae) and *Sitophilus oryzae* (L.) (Coleoptera: Curculionidae). *Journal of Stored Products Research*.

[11] El-Lakwah FA, Darwish AA, Khaled OM (1992). Effectiveness of Dill seed powder on stored products insects. *Annals of Agricultural Science, Moshtohor*.

[12] Duarte-Almeida JM, Negri G, Salatino A (2004). Volatile oils in leaves of *Bauhinia* (Fabaceae Caesalpinioideae). *Biochemical Systematics and Ecology*.

[13] Romestaing C, Piquet MA, Bedu E (2007). Long term highly saturated fat diet does not induce NASH in Wistar rats. *Nutrition and Metabolism*.

[14] Barham D, Trinder P (1972). A colorimetric method for the determination of Creatinine in serum. *Analyst*.

[15] Reitman S, Frankel S (1957). A colorimetric method for the determination of serum glutamic oxalacetic and glutamic pyruvic transaminases. *American Journal of Clinical Pathology*.

[16] Wilkinson JH, Boutwell JH, Winsten S (1969). Evaluation of a new system for the kinetic measurement of serum alkaline phosphatase. *Clinical Chemistry*.

[17] Bancroft JD, Stevens A (1996). *Theory and Practice of Histopathological Techniques*.

[18] Tapondjou LA, Adler C, Bouda H, Fontem DA (2002). Efficacy of powder and essential oil from *Chenopodium ambrosioides* leaves as post-harvest grain protectants against six-stored product beetles. *Journal of Stored Products Research*.

[19] Ketoh GK, Koumaglo HK, Glitho IA (2005). Inhibition of *Callosobruchus maculatus* (F.) (Coleoptera: Bruchidae) development with essential oil extracted from *Cymbopogon schoenanthus* L. Spreng. (Poaceae), and the wasp *Dinarmus basalis* (Rondani) (Hymenoptera: Pteromalidae). *Journal of Stored Products Research*.

[20] Park IK, Lee SG, Choi DH, Park JD, Ahn YJ (2003). Insecticidal activities of constituents identified in the essential oil from leaves of *Chamaecyparis obtusa* against *Callosobruchus chinensis* (L.) and *Sitophilus oryzae* (L.). *Journal of Stored Products Research*.

[21] Negahban M, Moharramipour S, Sefidko F (2006). Chemical composition and insecticidal activity of *Artemisiascoperte* essential oil against three Coleoptera stored-product insects. *Journal of Asia-Pacific Entomology*.

[22] Rozman V, Kalinovic I, Korunic Z (2007). Toxicity of naturally occurring compounds of Lamiaceae and Lauraceae to three stored-product insects. *Journal of Stored Products Research*.

[23] Ogendo JO, Kostyukovsky M, Ravid U (2008). Bioactivity of *Ocimum gratissimum* L. oil and two of its constituents against five insect pests attacking stored food products. *Journal of Stored Products Research*.

[24] López MD, Jordán MJ, Pascual-Villalobos MJ (2008). Toxic compounds in essential oils of coriander, caraway and basil active against stored rice pests. *Journal of Stored Products Research*.

[25] Ragasa CY, Hofileña JG, Rideout JA (2002). New furanoid diterpenes from *Caesalpinia pulcherrima*. *Journal of Natural Products*.

[26] Gordon WP, Forte AJ, McMurtry RJ (1982). Hepatotoxicity and pulmonary toxicity of pennyroyal oil and its constituent terpenes in the mouse. *Toxicology and Applied Pharmacology*.

[27] Miyazawa M, Watanabe H, Kameoka H (1997). Inhibition of acetylcholinesterase activity by monoterpenoids with a p-menthane skeleton. *Journal of Agricultural and Food Chemistry*.

[28] Lee SE, Choi WS, Lee HS, Park BS (2000). Cross-resistance of a chlorpyrifos-methyl resistant strain of *Oryzaephilus surinamensis* (Coleoptera: Cucujidae) to fumigant toxicity of essential oil extracted from *Eucalyptus globulus* and its major monoterpene, 1,8-cineole. *Journal of Stored Products Research*.

[29] Shaaya E, Kostjukovski M, Eilberg J, Sukprakarn C (1997). Plant oils as fumigants and contact insecticides for the control of stored-product insects. *Journal of Stored Products Research*.

[30] Wei LW, Wei M, Bing-yu Z, You-chen D, Feng I (2008). Antagonistic activities of volatiles from four strains of *Bacillus* spp. and *Paenibacillus* spp. against soil-borne plant pathogens. *Agricultural Sciences in China*.

